# Expanding Theranostic Radiopharmaceuticals for Tumor Diagnosis and Therapy

**DOI:** 10.3390/ph15010013

**Published:** 2021-12-22

**Authors:** Cristina Barca, Christoph M. Griessinger, Andreas Faust, Dominic Depke, Markus Essler, Albert D. Windhorst, Nick Devoogdt, Kevin M. Brindle, Michael Schäfers, Bastian Zinnhardt, Andreas H. Jacobs

**Affiliations:** 1 European Institute for Molecular Imaging, University of Münster, D-48149 Münster, Germany; faustan@uni-muenster.de (A.F.); depke@uni-muenster.de (D.D.); schafmi@uni-muenster.de (M.S.); bastian.zinnhardt@roche.com (B.Z.); 2 Roche Innovation Center, Early Clinical Development Oncology, Roche Pharmaceutical Research and Early Development, CH-4070 Basel, Switzerland; christoph_michael.griessinger@roche.com; 3 Department of Nuclear Medicine, University Hospital Münster, D-48149 Münster, Germany; 4 Department of Nuclear Medicine, University Hospital Bonn, D-53127 Bonn, Germany; markus.essler@ukbonn.de; 5 Department Radiology & Nuclear Medicine, Amsterdam UMC, Vrije Universiteit, De Boelelaan 1117, 1081HV Amsterdam, The Netherlands; ad.windhorst@amsterdamumc.nl; 6 In Vivo Cellular and Molecular Imaging Laboratory, Vrije Universiteit Brussel, B-1090 Brussel, Belgium; ndevoogd@vub.be; 7 Cancer Research UK Cambridge Institute, University of Cambridge, Cambridge CB2 ORE, UK; kmb1001@cam.ac.uk; 8 Biomarkers and Translational Technologies, Pharma Research and Early Development, F. Hoffmann-La Roche Ltd., CH-4070 Basel, Switzerland; 9 Department of Geriatrics and Neurology, Johanniter Hospital, D-53113 Bonn, Germany; 10 Centre of Integrated Oncology, University Hospital Bonn, D-53127 Bonn, Germany

**Keywords:** theranostics, tumor, gene therapy, cell-based therapy, molecular imaging, positron emission tomography, radiopharmaceuticals

## Abstract

Radioligand theranostics (RT) in oncology use cancer-type specific biomarkers and molecular imaging (MI), including positron emission tomography (PET), single-photon emission computed tomography (SPECT) and planar scintigraphy, for patient diagnosis, therapy, and personalized management. While the definition of theranostics was initially restricted to a single compound allowing visualization and therapy simultaneously, the concept has been widened with the development of theranostic pairs and the combination of nuclear medicine with different types of cancer therapies. Here, we review the clinical applications of different theranostic radiopharmaceuticals in managing different tumor types (differentiated thyroid, neuroendocrine prostate, and breast cancer) that support the combination of innovative oncological therapies such as gene and cell-based therapies with RT.

## 1. Introduction

The name “theranostics” reflects the combination of a therapeutic marker with a diagnostic tool to enable therapy and visualization simultaneously or sequentially. Theranostics in nuclear medicine, or radiopharmaceutical theranostics, is currently one of the leading medical fields promoting the development of theranostics, with the potential to make an important contribution to cancer therapy. Radioligand theranostics (RT) rely on the combination of disease-related biomarkers (enzyme, receptor, transporter) with the delivery of a radioactive compound that can be visualized by molecular imaging (MI), including planar scintigraphy, positron emission tomography-computed tomography (PET-CT), positron emission tomography-magnetic resonance imaging (PET-MRI) and single-photon emission computed tomography (SPECT).

MI is essential in the clinical translation of experimental paradigms by allowing the safe and repeated non-invasive assessment of the spatiotemporal distribution of target expression in vivo, allowing characterization of the diseased target tissue and providing imaging readouts of therapy response. MI employs radiopharmaceutical agents to target a specific disease-related biomarker (gene or protein expression) and visualize its distribution within the organism through the emission of radioactive particles. The imaging and therapeutic applications of an isotope depend on its particle emission: gamma (γ)-ray emitters such as technetium-99m (^99m^Tc, T_1/2_ = 6.0 h), iodine-123 (^123^I, T_1/2_ = 13.2 h) and positron emitters such as carbon-11 (^11^C, T_1/2_ = 20.4 min), fluorine-18 (^18^F, T_1/2_ = 109.6 min) and gallium-68 (^68^Ga, T_1/2_ = 1.13 h) are commonly used for diagnostic purposed using SPECT and PET imaging respectively, while α- and β- emitters are mostly, but not exclusively, used for radionuclide therapy [1]. The latter emit high-energy particles able to induce tissue and DNA damage and subsequent cell death. The α-emitters such as bismuth-213 (^213^Bi, T1/2 = 46 min), actinium-225 (^225^Ac, T_1/2_ = 9.9 days), lead-212 (^212^Pb, T_1/2_ = 10.6 h) and thorium-227 (^227^Th, T_1/2_ = 18.7 days) and β- emitting radionuclides such as lutetium-177 (^177^Lu, T_1/2_ = 6.7 days) or iodine-131 (^131^I, T_1/2_ = 8.0 days) show fractional γ-ray emission and, therefore, enable therapy and imaging simultaneously. However, their use could lead to unnecessarily high radiation exposure in patients when used for diagnostic purposes only (e.g., pre-treatment evaluation) and poor image quality due to low abundance of the γ-rays. Therefore, theranostic pairs, with similar structures and a matching pair of radionuclides, better serve diagnostic purposes by lowering radiation burden and achieving better image quality, ultimately differentiating the purely diagnostic from the therapy tracking radioisotope.

The α-emitting radionuclides have gained interest since α-particles have much higher linear energy transfer (LET) than the low LET β-particles and cause more tissue damage in a shorter range, and therefore spare the surrounding non-tumor tissue [2]. They are currently developed for clinical applications to enhance the anti-tumor effect of β-radiation in metastatic neuroendocrine tumors [3,4,5].

**Figure 1:**
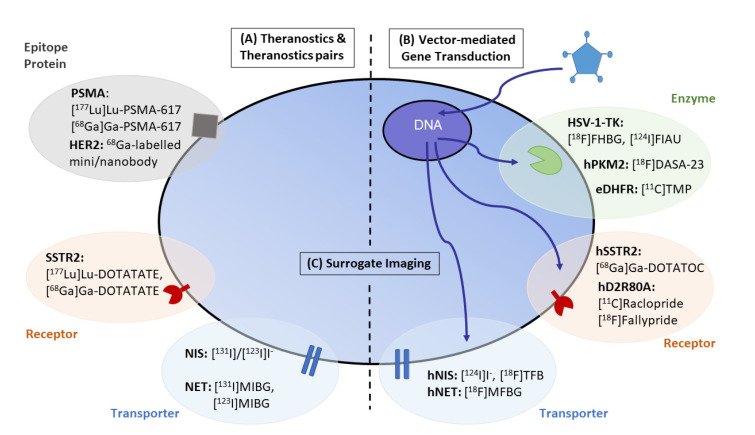
Overview of theranostic applications in oncology. (**A**) Theranostic applications in oncology have gained importance in the management of remnant tumors using cancer-type specific biomarkers, including SSTR2-positive or NET-positive neuroendocrine tumors, NIS-positive differentiated thyroid tumors and PSMA-positive prostate cancer. (**B**) The combination of vector-mediated gene transduction with the corresponding PET/SPECT-CT imaging probe not only benefits tracking of the efficiency of gene transduction but also establishes the fundamental principles to enlarge the field of theranostic applications by inducing the expression of an enzyme, receptor or transporter targeted by the corresponding theranostic radiopharmaceuticals. (**C**) Surrogate imaging relies on the visualization by molecular imaging of the downstream effects (metabolism, proliferation, associated inflammation) of a gene or cell-based therapy paradigm. Image modified from Jacobs et al. (2021) [6].

Different theranostic strategies in oncology have been developed: (i) direct visualization and quantification of target expression using a single radiolabelled compound for diagnosis and therapy without altering expression of the target; (ii) theranostic pairs combining two radiopharmaceuticals that share the same structure and target but are differentially labelled with matching radioisotope pairs that allow diagnosis and therapy separately; (iii) indirect imaging using reporter gene technology; and (iv) imaging downstream effects of gene and cell-based therapies. These strategies will be illustrated in the following sections (Figure 1).

## 2. Direct Visualization of Target Expression and Therapy Response

Direct MI of target expression involves direct binding or chemical interaction between the imaging probe and the disease-specific molecular target. Therefore, systemic treatment with a radioactively labeled target-specific tracer aims to selectively and directly hit cells associated with the disease pathology e.g., tumor cells [7]. Tracer accumulation and localization are directly related to the interaction of the tracer with its target, which in most cases is a receptor, transporter, enzyme or cell surface protein [6] (Figure 1A).

One of the most well-known theranostic applications is the use of radioiodine for the diagnosis and therapy of differentiated thyroid cancer. Iodine is taken up by the thyroid gland through the sodium iodide (NaI) membrane symporter (NIS), which symports Na^+^ together with various negatively charged ions to produce thyroid hormones. Radioactive iodide ([^131^I]NaI) is trapped by thyroid follicular cells, establishing the basis of radioiodine therapy (RAI) [8]. RAI is recommended for metastatic NIS-positive differentiated thyroid cancer, such as papillary or follicular thyroid cancers that show high levels of circulating thyroid-stimulating hormone (TSH), which stimulates iodine uptake. After surgical resection, ^131^I-mediated whole-body scintigraphy (WBS) allows visualization of the remnant thyroid tissue and metastases following surgical resection [9,10], allowing personalized patient management. RAI is recommended in case of remnant tissue with a high risk of recurrence since it was shown to improve overall survival and decrease recurrence [11]. Recently, ^131^I SPECT-CT has shown greater precision and accuracy over planar scintigraphy in detecting residual iodine in avid and non-avid cancer tissue and distant metastases, improving staging and patient management [12].

## 3. Theranostic Pairs

Diagnostic and therapeutic radiopharmaceuticals that access the same cellular or biological process with paired radiolabels are called theranostic pairs (Table 1). Among them, ^43/44^Sc/^47^Sc, ^64^Cu/^67^Cu, ^83^Sr/^89^Sr, ^86^Y/^90^Y, ^110^In/^111^In, ^124^I/^131^I, ^152^Tb/^161^Tb, ^152^Tb/^149^Tb, ^68^Ga/^177^Lu and ^90^Y/^177^Lu have been reported [13]. The first use of a theranostic pair was described using ^86^Y/^90^Y, which was applied in a patient with disseminated breast cancer bone metastases [14,15]. Other pairs have already found application in clinical research, while others are currently being developed. Frequently used in the clinics is the pairing of radionuclides of different elements, such as ^68^Ga for PET imaging and ^177^Lu as β^-^ emitter for therapy (e.g., coupled to ligands specific for somatostatin receptors (SSTR) or prostate-specific membrane antigen (PSMA)).

Furthermore, there is an increasing interest of nuclear physicians and the pharma industry in α-emitters which are available for theranostic approaches. ^211^At, ^223^Ra, ^213^Bi/^212^Bi, ^225^Ac or ^227^Th are examples of alpha emitters that are currently explored in the clinic [16]. Another interesting isotope is ^212^Pb, an in vivo generator of the alpha-emitting isotope ^212^Bi. As potential theranostic pairing, ^68^Ga and ^225^Ac or ^203^Pb and ^212^Pb were described for theranostic approaches with α-emitters [17,18,19].

### 3.1. Thyroid Cancer

A well-known example is the theranostic pair ^123^I/^131^I, which allows high-quality SPECT/CT-based dosimetry for pre- and post-therapeutic assessment of differentiated thyroid cancer. While ^131^I SPECT-CT imaging showed better accuracy than scintigraphy, image quality is limited by the low abundance of the gamma photons. ^123^I-mediated SPECT-CT imaging has increased in popularity in pre-therapy imaging due to improved resolution, higher sensitivity and lower radiation exposure compared to ^131^I [20]. The use of ^124^I, a potential diagnostic agent for PET imaging, is still limited in its use due to high cost and low availability. However, in a recent clinical trial, ^124^I-mediated PET imaging was proven safe and effective in finding metastatic lesions and is currently tested being as a predictor of exposure of metastases to ^131^I-mediated irradiation (NCT00673010).

### 3.2. Neuroendocrine Tumors

The therapeutic strategy to treat neuroendocrine tumors depends on the origin, tumor differentiation, grade, and stage. Despite favourable outcomes after surgical resection, recurrence and metastases are often observed and could therefore benefit from radionuclide therapy. While they form a diverse group of neoplasms, neuroendocrine tumors express specific molecular targets that can be exploited by MI for both diagnostic and therapeutic purposes.

#### 3.2.1. Norepinephrine Transporter (NET)

The agent [^131^I]metaiodobenzylguanidine ([^131^I]MIBG) allows visualization and concomitant therapy of neuroendocrine tumors that express the norepinephrine transporter (NET) [10]. [^131^I]MIBG radiotherapy is recommended in case of relapsed/refractory neuroblastoma (stage III or IV), inoperable metastatic phaeochromocytoma, paraganglioma, carcinoid tumors or recurrent medullary thyroid carcinoma [21]. NET is a Na^+^/Cl^−^ dependent transporter involved in the reuptake of extracellular norepinephrine (and also dopamine) by neuroendocrine cells, thereby regulating neurotransmitter concentration in the synaptic cleft. As with the endogenous neurotransmitters, [^131^I]MIBG is trapped by active endocytosis or by passive diffusion in NET-expressing cells of the sympathetic nervous system and either remains in the cytoplasm or is stored in neurosecretory granules [22,23]. Diagnostic [^123^I]/[^131^I]MIBG imaging prior to radiotherapy allows detection of MIBG-avid tissue and prediction of the beneficial effect of [^131^I]MIBG therapy. Overall, the response rate to [^131^I]MIBG therapy in pheochromocytomas and paragangliomas increases with dose: the response rate increases with a higher radiation dose than 18.5 MBq or with repeated exposure, leading to a progression-free survival of up to 85 months [24,25,26].

[^123^I]MIBG offers better image quality with planar/SPECT imaging [27], and has a prognostic value that can be used to guide patient management: patients with stage 4 neuroblastoma and [^123^I]MIBG positive metastases have a better prognosis and longer survival after therapy, while [^123^I]MIBG negativity indicates a worse outcome [23]. Overall, [^123^I]MIBG scintigraphy showed a sensitivity of 88% and a specificity of 83% in a prospective trial to test diagnostic performance in neuroblastoma [28]. Comparable results were observed for [^123^I]MIBG imaging in the diagnosis of phaeochromocytoma and paraganglioma [29]. A fluorinated analogue, ^18^F-metafluorobenzylguanidine ([^18^F]MFBG), which shows greater contrast and detects additional lesions that are not observed with [^123^I]MIBG imaging, is currently been evaluated in a prospective study (NCT02348749) [30] as a possible alternative for PET imaging of NET-positive neuroblastoma [23,31]. An ongoing clinical trial is evaluating the potential and feasibility of [^18^F]MFBG PET imaging in patients with neural crest and neuroendocrine tumors in comparison to [^123^I]MIBG imaging (NCT04258592).

#### 3.2.2. Somatostatin Receptors

The somatostatin receptors (SSTRs including SSTR1, 2A and B, 3, 4, and 5) are also overexpressed in most differentiated neuroendocrine tumors where the somatostatin ligand suppresses tumor cell proliferation, survival, and angiogenesis. Therefore, radionuclide-labeled derivatives of somatostatin receptor agonists and antagonists have been developed that permit the non-invasive imaging of SSTRs [32,33,34]. Traditionally, SSTR-positive tumors have been diagnosed using [^111^In]In-pentetreotide. Over the past decade, a plethora of diagnostic agents has been investigated for PET imaging. The increased interest in imaging SSTRs was triggered by the establishment of [^68^Ga]Ga-DOTATATE, [^68^Ga]Ga-DOTATOC and [^68^Ga]Ga-DOTANOC tracer imaging, which showed high sensitivity (82–97%) and specificity (80–92%) for detecting neuroendocrine tumors [35] and improved properties over the [^111^In]In-pentetreotide SPECT tracer [36,37]. [^68^Ga]Ga-DOTATATE shows higher selectivity for SSTR2, [^68^Ga]Ga-DOTATOC binds SSTR2 and SSTR5 while [^68^Ga]Ga-DOTANOC has a wider binding profile, including SSTR2, SSTR3 and SSTR5 [38].

The most promising and currently used theranostic paradigm in neuroendocrine tumors employs [^177^Lu]Lu-DOTATATE (Lutathera^®^). Lutathera^®^ was approved by the EMA in 2017 and the FDA in 2018 for the treatment of SSTR-positive gastroenteropancreatic (GEP) neuroendocrine tumors [39]. Inclusion criteria for therapy are based on MI: the patient must present a metastatic, inoperable differentiated NET that shows sufficient [^111^In]In-pentetreotide or ^68^Ga-labeled somatostatin receptor tracer uptake in the tumor tissue [40].

The Neuroendocrine Tumors Therapy (NETTER-1) study published the first phase 3 trial using [^177^Lu]Lu-DOTATATE to treat midgut NETs (NCT01578239) [41,42]. After a first phase of 18-month treatment period, patients entered the long-term follow-up phase (76.3 months vs. 76.5 months) and results were recently released [43]. [^177^Lu]Lu-DOTATATE therapy combined with standard of care treatment (7.4 GBq every 8 weeks + 30 mg octreotide LAR) compared to high-dosage octreotide (60 mg every 4 weeks) resulted in markedly longer progression-free survival (HR = 0.18, *p* < 0.0001), higher overall survival (48 months vs. 36.3 months, HR: 0.84, *p* = 0.3) and higher response rate (18 vs. 4% for the octreotide arm) in NET patients. In both arms, assessment of long-term safety issues revealed 1.8% myelodysplastic syndrome in [^177^Lu]Lu-DOTATATE arm, and no significant effect in nephrotoxicity (5.4 vs. 3.6%) [41,42].

While β-emitting peptide receptor radioligand-therapy (PRRT) has been shown to increase the progression-free survival, the response rate remains low. The α-PRRT including [^225^Ac]Ac-DOTATATE; [^225^Ac]Ac-DOTATOC or [^212^Pb]Pb-DOTAMTATE (Alphamedix) are currently under clinical development in patients with β-radiation refractory metastatic NET to enhance the anti-tumor effect or overcome therapy resistance. They have already brought clinical benefits and long-time survival to patients with metastatic NETs [4,5,44]. In a prospective study (median follow-up of 8 months), 75% of patients with stable or progressive metastatic GEP-NETs treated with [^225^Ac]Ac-DOTATATE showed partial remission [3]. A similar anti-tumor effect was observed after [^225^Ac]Ac-DOTATOC α- PRRT, including reduced tumor volume and fewer lesions [4]. Correct dosage and safety were recently investigated: hematotoxicity could be avoided with activities of 20 MBq every 4 months [5].

### 3.3. Prostate Cancer

Most prostate cancers (PCa) overexpress the prostate-specific membrane antigen (PSMA), a transmembrane glycoprotein, on the PCa cell membrane. PSMA is also found in the neovasculature of many carcinomas. Overexpression of PSMA on prostate epithelial cells is associated with malignant, castration-resistant PCa and tumor aggressiveness. Since PSMA is internalized after binding to the ligand, it provides an excellent target for radionuclide therapy, inducing direct DNA damage, reducing the risk of unspecific radiation, and improving the tumor-to-background uptake ratio.

Many small-molecule PSMA-targeting radiopharmaceuticals are available. [^123^I]MIP-1072 and [^123^I]MIP-1095 were the first to show clinical potential for imaging prostate cancer, and the replacement of ^123^I by ^131^I led to the first theranostics application, showing positive tumor response to therapy in 70% of the patients after a single dose [45,46]. The development of the highly specific PET tracer [^68^Ga]Ga-PSMA-11 represented a step forward in diagnosing recurrent PCa [47]. Other small molecules for PET imaging were investigated, such as [^68^Ga]Ga-PSMA-617 and [^68^Ga]Ga-PSMA-I&T, which were converted into ^177^Lu- or ^90^Y-labeled agents for therapeutic purposes [35,48]. The PET tracer [^68^Ga]Ga-PSMA-617 PET tracer shows the highest uptake in the kidneys and salivary glands and accumulates in the metastatic lesions at 2–3 h post-injection. Toxicity in salivary glands and kidneys is dose-limiting, inducing xerostomia in patients, but theranostics support the safety strategy as it can identify organs at risk.

Its counterpart, [^177^Lu]Lu-PSMA-617, binds to PSMA with high affinity in castration-resistant metastatic PCa and low toxicity profile. It reduced pain and gave a high positive response rate (more than 50%) in those patients who progressed after standard treatment [48,49,50,51,52]. Overall, there is compelling evidence that [^177^Lu]Lu-PSMA-617 has promising anti-tumor activity in men with resistant and metastatic PCa. Although planar scintigraphy with [^177^Lu]Lu-PSMA-617 allowed accurate follow-up, [^68^Ga]Ga-PSMA-11 PET imaging is preferred for patient selection and final response assessment together with serum prostate-specific antigen (PSA) level as a well-established surrogate marker of therapy efficiency [51]. Comparative clinical trials that aim to determine the activity and safety of the theranostic agent compared to other metastatic cancer treatments are currently ongoing (NCT03511664, NCT03392428).

Despite good tolerability and positive response rate to [^177^Lu]Lu-PSMA-617 PRRT, a considerable number of patients were not responsive to β-emitters PRRT and showed adverse treatment effects such as xerostomia or hematologic toxicity. Currently [^225^Ac]Ac-PSMA-617 is tested in clinical trials and showed already durable complete responses in metastatic prostate cancer in heavily pretreated patients but also chemotherapy-naïve patients [5,53,54]. A retrospective study by Kratochwil and colleagues (2018) reported on the outcomes of [^225^Ac]Ac-PSMA-617 PRRT as a salvage last-line therapy on 38 eligible heavily pretreated advanced PCa patients [54]. At 8 and 16 weeks, PSA levels declined more than 50% in 63% of the patients, and complete remission was observed in 13% of the patients, leading to a better PSA response rate than [^177^Lu]Lu-PSMA-617 [17,55]. An early case report of two advanced-stage prostate cancer patients showed complete remission after [^225^Ac]Ac-PSMA-617 PRRT with no relevant treatment adverse effects, overall supporting that [^225^Ac]Ac-PSMA-617 could represent a new salvage therapy in advanced PCa [53]. Additionally, a pilot study reported on the use of [^225^Ac]Ac-PSMA-617 as a first-line therapy in chemotherapy-naive patients with advanced prostate cancer, in which good anti-tumor response was observed in 16/17 patients following two or three cycles of [^225^Ac]Ac-PSMA-617, with 11/17 patients showing complete resolution of all metastatic lesions [56].

New in the field of immune-targeting of cancers and PSMA-targeting therapies, nanobodies and nanobody-mediated targeted-radionuclide therapies have gained interest because of their high antigen specificity, low-immunogenicity, and small size, which allows specific imaging and the design of therapies that target antigens on hidden epitopes. However, their use is currently limited to preclinical research [57,58,59].

### 3.4. Alternative Approaches following the Classical Theranostic Approach

A plethora of additional novel theranostic and radiotherapy approaches are currently in preclinical and clinical development, focusing on different targets apart from PSMA and SSTR. In this section, we would like to focus on a few additional promising targets which could offer a broad applicability for theranostics in the future.

#### 3.4.1. Human Epidermal Growth Factor Receptor Type 2

The Human Epidermal Growth Factor Receptor type 2 (HER2), a transmembrane tyrosine kinase receptor, is overexpressed in breast, ovarian and gastric cancers. Signalling through the dimerization of HER2 promotes tumor cell proliferation and inhibition of apoptosis. The monoclonal antibodies trastuzumab (Herceptin^®^), pertuzumab (Perjeta^®^) and the antibody-drug conjugate trastuzumab emtansine (Kadcyla^®^) have paved their way in HER2-positive breast cancer (BCa) therapy by prolonging patients’ survival. Trastuzumab prevents the receptor’s ligand-independent dimerisation and induces its degradation, stimulating the immune system to recognize and eliminate HER2-overexpressing cells. Its combination with pertuzumab prevents ligand-dependent dimerization and allows to overcome trastuzumab resistance by complementary action.

Radiolabeled [^89^Zr]Zr-trastuzumab was used for imaging approaches and ^227^Th-labeled HER2 (NCT04147819) antibodies are currently developed for therapeutic purposes [60,61]. Laforest and colleagues conducted the first-in-human ^89^Zr-trastuzumab PET imaging clinical trial in patients with metastatic BCa [61]: great tumor-to-non tumor contrast was achieved 5+/−1 days post injection in HER2-positive BCa. However, high uptake and long residence time were observed in the liver, and brain metastases were only detected with a compromised blood-brain barrier. Several studies concluded on the potential of [^89^Zr]Zr-trastuzumab to visualize HER2-positive metastases from HER2-negative primary cancer and lesions that do not respond to treatment [62,63]. Trastuzumab and derivatives have been labelled with β^-^ emitting radionuclides for theranostic purposes [64]: a patient study showed [^177^Lu]Lu-DOTA-trastuzumab uptake in primary and metastatic BCa lesion and no uptake in HER2-negative sites, attesting on its specificity [64]. However, full antibodies as carrier molecules have limitations for imaging, e.g., detection sensitivity due to their long plasma half-life. In line, the long plasma half-life could also cause dose-limiting haematological toxicities when coupled to a therapeutic isotope.

A novel theranostic approach is currently in clinical development which utilizes HER2-specific single domain antibodies (sdAb). [^131^I]GMIB-anti-HER2-VHH1 is the lead candidate in the therapeutic development [65]. Phase I trial (NCT04467515) demonstrated the safety and imaging potential of the theranostic agent in BCa patients: first data from low dose cohorts showed that administration of [^131^I]GMIB-anti-HER2-VHH1 was well-tolerated, safe, and showed tracer uptake in HER2-positive metastatic lesions [66,67]. A subsequent phase I/II study includes the dose escalation to assess the therapeutic window. Additionally, PET imaging with [^68^Ga]Ga-HER2-sdAb ([^68^Ga]Ga-NOTA-HER2) was successfully conducted in clinical trials and a Phase II is currently ongoing [66,68].

#### 3.4.2. Fibroblast Activating Protein

A potential target for a new theranostic approach with very broad applicability is the fibroblast activating protein (FAP) expressed by cancer-associated fibroblasts in the tumor stroma of many tumor indications. Moreover, cancer cells can express FAP in some indications, e.g., sarcoma or melanoma.

Recently, a ^68^Ga-labelled FAP-inhibitor (FAPi-04) was developed by the University of Heidelberg which is highly suitable to detect FAP-containing tumor lesions by PET imaging [69]. In further studies, it was demonstrated that the FAPi tracer is capable to visualize tumor lesions in 28 different types of cancer with high sensitivity and image quality [70]. First compassionate use studies with ^90^Y,^177^Lu or ^153^Sm-labeled FAPi-derivatives (FAPi-46, DOTA.SA.FAPi, FAPi-04) were conducted in breast cancer, sarcoma and pancreatic cancer patients. The therapies were well tolerated and the first signs of clinical responses, such as stable disease or shrinkage of individual metastases as well as reduction of clinical symptoms (e.g., pain) were observed [71,72,73,74].

#### 3.4.3. Chemokine Receptor C-X-CR-4

The chemokine receptor C-X-CR-4 (CXCR4) is an important receptor for the migration of stem cells. Most of the tumor cells also express CXCR4 and the receptor supports proliferation, angiogenesis, survival, and metastasis formation. The available theranostic approach with [^68^Ga]Ga-Pentixafor and [^177^Lu]Lu/[^90^Y]Y-Pentixather showed clinical benefit in combination with chemotherapy and autologous stem cell transfer in advanced multiple myeloma. Further clinical trials in lymphoma and multiple myeloma are ongoing [75,76].

More interesting approaches focusing on CA-IX, mesothelin, CD33 and alternative targets were nicely reviewed by Sgouros et al. [16] and Solnes et al. [77].

## 4. Theranostics Using Gene and Cell-Based Therapy

Gene therapy enables the targeted delivery of gene-based cassettes that facilitate the stable, sustained and regulated expression of biological agents in the diseased tissue. This can be used to replace the function of a defective gene leading to the cure of the pathological genotype or which produces a targeted molecular intervention leading to functional improvement and hence the clinical status of the patient [6,78]. Thousands of clinical trials utilizing gene therapy have been designed in the past 15 years to treat inherited disorders and a wide variety of acquired diseases [78,79], with a large majority applied to cancer therapy. Many cancer gene therapy approaches have been developed: (i) replacement of tumor suppressor genes; (ii) gene or RNA targeting approaches; (iii) drug sensitization by transduction with suicide genes; (iv) genetic immunotherapy; (v) transfer of genes interfering with the biological program of tumor growth; (vi) oncolytic viral therapy; and (vii) CAR T-cell therapy [6]. The success of clinical gene therapy is currently judged based on clinical observations and advanced molecular readouts, including insertional vector analysis. To move gene and cell-based therapies forward, the technologies being used in the field of MI will be highly beneficial. Indeed, MI not only allows the non-invasive assessment of the disease-specific and –driving molecular events in vivo but also enables quantitative assessment of transduced cells and gene, allowing therapy monitoring and the refinement of treatment protocols [6].

### 4.1. Tools for Gene and Cell-Based Therapy

Gene therapy uses reporter gene systems encoding proteins, enzymes (e.g., HSV-1-TK, luciferase), transporters (e.g., hNIS), cell-surface receptors (e.g., hD2R, hSSTR2), an antigen (Her2, PSMA) or a fluorescent protein (e.g., eGFP) [6,80,81] (Figure 1B). Various methods were developed to deliver genes into the target tissue: non-viral vectors (plasmid DNA with or without carrier molecules, lipid nanoparticles), viral vectors (replication-competent oncolytic viruses or gene therapy viral vectors) or mesenchymal stem cells (MSCs) [82]. The reporter gene does not have a therapeutic role itself, but by coupling it to a therapeutic gene, the expression of the reporter gene reports indirectly on the expression of the therapeutic gene. Additionally, more than one gene could be inserted into the reporter construct to either improve therapy outcomes or allow visualization using different imaging methods, underlining the versatility of the reporter gene. In principle, these reporter genes could be used in vivo for the non-invasive assessment of gene therapy protocols. PET-based reporters are of particular interest due to their quantitative and translational value. There are many reports on the construction and validation of several multimodality bifusion and triple fusion reporter genes in living animals [83,84,85,86]. The most commonly used reporter genes for MI studies using radiolabeled probes and PET imaging are wild-type herpes simplex virus 1 thymidine kinase (HSV-1-tk) and mutant HSV-1-sr39tk [87]. Furthermore, seven human reporter genes with their corresponding imaging probes were reported, including hNIS, hNET, the human dopamine type 2 receptor (hD2R), hSSTR2, the human mitochondrial thymidine kinase type 2 (hTK2), the human deoxycytidine kinase double mutant (hdCKDM), hPSMA [80]. In the next section, a few examples of reporter genes will be discussed (Table 2) (Figure 1B).

### 4.2. Gene and Cell-Based Therapy for Theranostic Applications

Indirect imaging strategies using reporter gene technology involve pre-targeting components that function as molecular-genetic sensors. Imaging the activity of the reporter gene product, or additional genes proportionally co-expressed with the reporter gene, through (i) the level of reporter probe accumulation or (ii) the level of emitted particles provides direct information that reflects the level of reporter gene expression and indirect information on the level of endogenous signaling and transcription factors that drive reporter gene expression [6,83,88].

#### 4.2.1. Enzyme Gene Reporter: HSV-1-tk Imaging Using [^18^F]FHBG or [^124^I]FIAU

**Figure 2:**
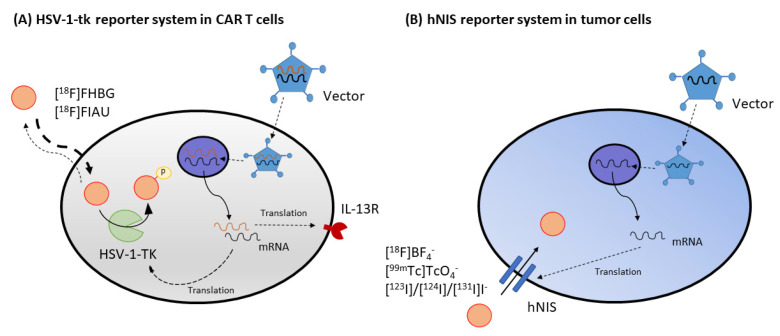
Successful reporter systems combined with molecular imaging are applied in clinical trials. (**A**) Keu et al. (2017) reported tumor-associated CD8^+^ engineered T lymphocytes expressing both HSV1-tk and interleukin-13 (IL-13) zetakine domain, a chimeric antigen receptor, could be tracked longitudinally by [^18^F]FHBG imaging in patients with high-grade glioma [96]. (**B**) Measles virus and vesicular stomatitis virus expressing hNIS were tested in cancer patients. Assessment of clinical outcomes included [^18^F]BF_4_^−^ PET or [^99m^Tc]TcO_4_^−^ SPECT imaging.

Enzyme-based reporters rely on the metabolic entrapment of a radiotracer using an enzyme not expressed or abundant in humans. A well-known example is the herpes simplex virus 1 thymidine kinase (HSV-1-tk) which has been used extensively as a reporter system in conjunction with radiotracers such as [^18^F]FHGB or [^124^I]FIAU for visualization (Figure 2A). This gene can be used simultaneously as a marker gene by administration of its radiolabeled substrates or as a therapeutic suicide gene by administering a prodrug. HSV-1 encodes a thymidine kinase that converts thymidine into its phosphorylated form and phosphorylates various prodrugs such as acycloguanosines (ganciclovir, acyclovir and penciclovir), producing monophosphorylated analogs that are then converted into triphosphorylated forms by cellular kinases. These prodrugs kill cells by either blocking DNA synthesis or causing chain termination during DNA replication, ultimately inducing the death of proliferating cells. This mechanism establishes the so-called suicide gene therapy paradigm [89]. Additionally, HSV-1-tk can also phosphorylate [^18^F]FHBG and 2′-fluoro-nucleoside analogs of thymidine such as [^124^I]FIAU, trapping these imaging tracers within the cell.

The HSV-1-tk system has been further elaborated in the past 15 years with regard to (i) the assessment of anti-cancer therapy paradigms [89,90,91,92], employing tumor-specific promoters [93,94], cancer-directed T-cells [95,96], transduced mesenchymal stem cells [97,98,99,100] or bone marrow-derived tumor-infiltrating progenitor cells; (ii) the visualization of tumor burden [101] and the primary anti-tumor immune response [102].

In experimental glioblastoma gene therapy, MI using [^18^F]FLT supported the identification of viable target tissue, which may benefit from HSV-1-tk gene therapy. Additionally, the same study localized the transduced tissue dose of HSV-1 amplicon vector-mediated therapeutic gene expression using in vivo [^18^F]FHBG PET imaging [90]. More recently, monitoring specific cell types and progeny in a non-invasive way has been achieved by combining Cre/lox-assisted cell fate mapping with a thymidine kinase reporter gene for PET imaging [95]. Platelets, T-cells and cardiomyocytes could be visualized by [^18^F]FHBG PET imaging. There have been many trials of mesenchymal stem cells (MSCs) as cell-based drug delivery systems in which tumors homing and assessment of their therapeutic effects were monitored by transducing the cells with HSV-1-tk to allow their visualization using PET [97,103]. One of the most important clinical applications of HSV-1-tk in CAR-T cells was published by Keu et al. (2017), which demonstrated that tumor-associated CD8^+^ engineered T lymphocytes expressing both HSV1-tk and interleukin-13 (IL-13) zetakine chimeric antigen receptor (CAR) could be tracked longitudinally by [^18^F]FHBG imaging in patients with high-grade glioma [96] (Figure 2A). The sensitivity to detect the cells in the brain with [^18^F]-FHBG was high due to a low background signal. Taken together, these studies indicated that the development of brain cancer cell-based therapies and immunotherapies could benefit greatly from a constitutive-promoter-driven reporter gene system to enable longitudinal monitoring of specific cell populations.

There remain, however, some major limitations in the successful application of gene therapy in cancer patients including (i) heterogeneity of the tumor tissue; (ii) limited transduction efficiency; and (iii) i.v. injection of vectors that give constitutive and widespread expression. For example, in prostate cancer, the use of a prostate-specific vector system decreased systemic toxicity while retaining tumor-killing capacity when compared to a constitutive unrestricted HSV-1-tk vector approach [93]. Therefore, tissue-restricted expression of a therapeutic or cytotoxic gene should achieve a superior therapeutic index over the unrestricted method. Nevertheless, HSV-1-tk still has many properties that make it suitable to treat cancer including its neurotropism, high transduction efficiency and its capacity to hold larger transgenes when compared to other vector systems [104]. Among the clinical trials using HSV-1-tk, the safety and efficacy of HSV-1-tk (gene therapy) in combination with valacyclovir, radiotherapy and chemotherapy in newly diagnosed and recurrent glioblastoma multiforme and grade III astrocytoma are currently being investigated (NCT03596086, NCT03603405). However, the GEN2 directed cancer immunotherapy trial is the only one that includes monitoring of HSV-1-tk-m2 expression using [^18^F]FHBG PET imaging (NCT04313868) while its use to monitor HSV-1-tk gene therapy in humans has already been validated [105], including the use of [^124^I]FIAU as the reporter probe [92].

#### 4.2.2. Receptor Gene Reporter: HSSTR2 Imaging Using [^68^Ga]Ga-DOTATOC, [^90^Y]Y-DOTATATE or [^177^Lu]Lu-DOTATATE

The human somatostatin receptor has also been used as a preclinical platform for imaging reporter gene therapy [106]. The human somatostatin receptor subtype 2 (hSSTR2) has been used as (i) reporter gene for adenoviral (AdV)-mediated gene transduction into preclinical non-small cell lung cancer [107] and breast cancer models [108] and visualized using [^68^Ga]Ga-DOTATATE- and [^68^Ga]Ga-DOTATOC PET imaging [109,110]; (ii) in a theranostics paradigm employing [^90^Y]Y-DOTATOC in hSSTR2-transduced xenografts [111]; (iii) in oncolytic virus (OV) therapy approaches [112,113]; and (iv) to study the dynamics of CAR T-cell responses in a mouse model of thyroid cancer [114].

Recent clinical trials reporting on hSSTR2-positive tumors (i) assessed the diagnostic agents in different types of cancer that partly express SSTR2 (NCT04298541), (ii) optimized imaging protocols, and (iii) assessed the potential of [^177^Lu]Lu-DOTATATE in GEP-NET (NCT02936323) and SSTR2-positive breast cancer (NCT04529044). However, the combination of PET/SPECT imaging with SSTR2-based gene therapy has not yet been reported in humans.

Nevertheless, there have been preclinical gene therapy studies exploring hSSTR2 reporter-based imaging and therapy in SSTR negative or low-expressing SSTR tumors using various gene transfer technologies. hSSTR2 is a relatively short sequence that allows packaging of additional therapeutic transgenes, and has a human origin and therefore, does not elicit a host immune response, unlike the HSV-1-tk reporter gene system. Additionally, SSTR2 is a surface receptor and therefore does not require cell uptake of the tracer. The delivery of SSRT2 to tumors using lentiviral vectors or murine mesenchymal stem cells has been successful when these are given intratumorally, where transgene delivery and expression in xenografts was assessed using [^68^Ga]Ga-DOTATOC [111]. As a first step towards clinical translation of the hSSTR2 reporter-based imaging and therapy, the delivery of the hSSTR2 transgene in xenografts was combined with [^90^Y]Y-DOTATOC and showed delayed tumor growth, supporting further research as a therapeutic approach in cancer [111].

Additionally, hSSTR2-based reporter genes have been expanded using other delivery systems preclinically. Oncolytic viruses (OVs) are advantageous since systemic delivery will target OV to all tumor sites, including metastases. hSSTR2-expressing OV has shown a therapeutic effect of radioactivity accumulation in tumor cells of mice bearing subcutaneous colon cancer xenografts, with a higher concentration of radiotracer in the tumor infected with the SSTR2-expressing oncolytic virus compared to the tumor infected with the control virus, and has allowed non-invasive visualization of the spatial distribution of virus for up to 3 weeks post-viral injection using repeated injections of [^111^In]In-pentetreotide [113]. Additionally, hSSTR2 was used as a reporter to track T cell infiltration and expansion within anaplastic thyroid tumors [114]. Despite the low density of transfected cells, SSTR2-expressing cells were detectable with high sensitivity and specificity using [^68^Ga]Ga-DOTATOC PET imaging [114].

#### 4.2.3. Transporter Gene Reporter: Human Sodium/Iodide Symporter (hNIS): [^124^I]Iodide and [^18^F]TFB

The expression of the human thyroid sodium/iodide symporter (hNIS) in non-thyroidal tumor tissue has been investigated in a variety of tumor models [115,116] and has been employed to facilitate radioiodine therapy [116,117]. One of the major drawbacks of the system is that radiolabeled iodide is not metabolically trapped within virally transduced tissues resulting in a lack of retention in non-thyroidal tissues. Therefore, quantification of gene expression is hindered, and the use of hNIS as a suicide protein is limited. However, the lack of retention of radioactivity in non-thyroidal tissues can be overcome by co-expression of both hNIS and thyroperoxidase (TPO), which catalyzes the iodination of proteins resulting in increased iodide retention and subsequently enhanced tumor cell apoptosis [118].

An interesting preclinical approach that is currently under evaluation as a tumor delivery vehicle relies on the intrinsic property of MSCs to migrate to the tumor tissue after irradiation. Engineered NIS-expressing MSCs resulted in tumor iodide uptake, which was monitored by ^123^I-mediated SPECT [119]. Further studies combined MSCs gene delivery with therapeutic irradiation (^131^I) and the control of the transgene expression using different promoters [115,120].

Various phase I/phase II clinical trials investigating NIS as a reporter and therapy gene have used virus-mediated NIS gene delivery for recurrent or refractory prostate cancer (NCT00788307), multiple myeloma combined with cyclophosphamide (NCT00450814, NCT02192775 NCT02192775) [121], and resistant ovarian cancer [122]. These and others are currently ongoing, with most of them evaluating the use of ^123^I-mediated SPECT imaging. Recently, other iodide analogs for PET imaging have emerged. A recent clinical trial performed a first-in-man comparative evaluation of the promising imaging probe [^18^F] tetrafluoroborate ([^18^F]BF_4_^−^ or [^18^F]TFB) for PET imaging [123] and [^99m^Tc]pertechnetate ([^99m^Tc]TcO_4_^−^) for SPECT imaging of hNIS expression in myeloma patients treated with Measles virus-NIS (MV-NIS) and endometrial cancer patients treated with vesicular stomatitis virus co-expressing interferon and hNIS (VSV-hINF-NIS) (NCT03456908) (Figure 2B). Results are pending. Overall, the theranostic hNIS reporter system may still benefit from improvements in the delivery system and radiotracer but it is on its way to a practical clinical theranostic approach.

#### 4.2.4. Transporter Gene Reporter: Human Norepinephrine Transporter (hNET): [^18^F]MFBG

The human norepinephrine transporter (hNET) reporter gene system is still limited to preclinical studies, but promising results using an oncolytic virus have been obtained [124]. Interestingly, a comparison of different gene-reporter/probe systems (hNET, hNIS, hdCKDM, HSV-1-TK) to determine the detection sensitivity for transduced T-cells showed that the human norepinephrine transporter (hNET) reporter combined with the [^18^F]MFBG PET imaging probe was the most sensitive system, which was capable of detecting 35–40 × 10^3^ T-cells at the site of T-cell injection in a preclinical model [125].

### 4.3. Surrogate Imaging 

**Figure 3:**
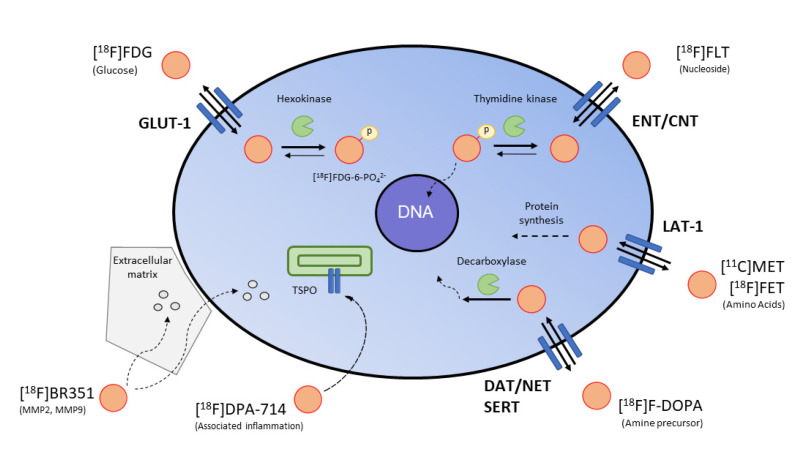
Surrogate imaging in oncology. Examples of key clinical PET tracers for imaging tumor metabolism, cell proliferation and tumor-associated inflammation.

Surrogate imaging relies on the visualization of the downstream effects induced by a gene therapy paradigm (Figure 3). In the context of cancer imaging, the destruction of pathological cells can be accompanied by an increase in cell death or a decrease in proliferation, which can be visualized by MI. As an example, [^18^F]FLT was used to assess the effects of gene therapy on tumor cell proliferation by performing PET imaging before and after gene therapy in glioblastoma [90,126,127]. Interestingly, the efficiency of gene transduction, as measured by [^18^F]FHBG PET, was correlated with the therapeutic effect on cell proliferation, as assessed by [^18^F]FLT PET imaging. Other imaging probes such as [^18^F]FET (amino acid metabolism, neovascularisation), [^18^F]FDG (glucose consumption) and [^18^F]FDOPA (amine precursor) can be used to characterize the metabolic state of the cancer cells in conjunction with vascular MR imaging [127,128]. Additionally, [^18^F]DPA-714 (inflammatory immune cells, translocator protein) and [^18^F]BR-351 (MMP2, MMP9) inform on the therapeutic effect of the gene therapy on tumor-associated inflammation [129,130].

## 5. Conclusions

The development of, and progress in, the field of theranostics demonstrate the benefits of combining nuclear medicine with different types of cancer therapies, including gene and cell-based therapies. With the increasing understanding of the inter- and intra-individual heterogeneity of tumors, theranostics turns out to be a smart therapeutic approach for personalized patient management. Successful clinical approaches such as LUTHATERA and PSMA-617 pave the way for further development in the field of theranostics. Expanding the field of theranostics using α-emitting radionuclides allowed to overcome some of the limitations of β- emitters with similar or enhanced anti-tumor activity. Additionally, there are now seven well-defined human reporter genes, including hNIS, hNET and hSSTR2 with complementary radiolabeled marker substrates available for clinical application; hNIS being the best characterized for theranostic applications and hNET being the most sensitive system to detect transduced cells. In this field, the use of HSV-1-tk in CAR-T cells together with the well-established [^18^F]FHBG tracer represents one of the most important clinical applications of the reporter system in humans. However, the use of gene and cell-based imaging strategies in theranostic approaches is still rare, partly due to the complex nature of clinical gene- and cell-based therapy protocols, the limitations in radiotracer production and the quantification of the imaging signal. However, it already provides a more comprehensive picture of underlying tumor biology, extending the range of theranostic applications, guiding personalized management, and supporting treatment refinement.

## Figures and Tables

**Table 1:** Examples of theranostic pairs.

Class	Diagnostic Agent	Therapeutic Agent	Target	Disease
Transporter	[^123^I]I^−^	[^131^I]I^−^	NIS (SLC5A5)	Differentiated thyroid cancer, hyperthyroidism
	[^123^I]MIBG, [^124^I]MIBG, [^18^F]MFBG	[^131^I]I-MIBG	NET (SLC6A2)	Neuroendocrine tumors, including neuroblastoma, pheochromocytomas, paragangliomas, medullary thyroid carcinoma
Cell-surface receptor	[^68^Ga]Ga-DOTATATE, [^68^Ga]Ga-DOTATOC, [^68^Ga]Ga-DOTANOC	[^111^In]In-pentetreotide, [^177^Lu]Lu-DOTATATE (LUTATHERA^®^), [^90^Y]Y-DOTATATE, [^225^Ac]Ac-DOTATATE, [^177^Lu]Lu-DOTATOC, [^90^Y]Y-DOTATOC, [^225^Ac]Ac-DOTATOC, [^212^Pb]Pb-DOTAMTATE	SSTRs	Neuroendocrine tumors, mostly gastroenteropancreatic tumor (GEP-NET)
Cell-surface protein	[^123^I]MIP-1072, [^123^I]MIP-1095, [^68^Ga]Ga-PSMA-11, [^68^Ga]Ga-PSMA-I&T, [^68^Ga]Ga-PSMA-617	[^131^I]I-MIP-1095, [^177^Lu]Lu-PSMA-I&T, [^177^Lu]Lu-PSMA-617, [^225^Ac]Ac-PSMA-617	PSMA	Metastatic prostate cancer
	[^89^Zr]Zr-trastuzumab, [^68^Ga]Ga-NOTA-HER2	[^177^Lu]Lu-trastuzumab and derivatives, [^131^I]GMIB-anti-HER2-VHH1	HER2	Breast, ovarian and gastric cancer

**Table 2:** Examples of gene reporter systems and their corresponding imaging probe.

Transgene	Imaging Probe	Clinical Trials/Applications
HSV-1-tk HSV-1-sr39tk	[^123^I]/[^124^I]/[^131^I]FIAU [^18^F]FHBG	Yes
hSSTR2	[^111^In]In-pentetreotide [^68^Ga]Ga-DOTATATE [^68^Ga]Ga-DOTATOC	No
hNIS	[^211^At]At^−^, [^188^Re]ReO_4_^−^, [^124^I]/[^131^I]I^−^, [^18^F]TFB	Yes
hNET	[^123^I]MIBG [^18^F]MFBG	No

## Data Availability

Not applicable.
